# The Top 100 Most Cited Articles on Epilepsy Surgery: A Bibliometric Analysis of the Literature

**DOI:** 10.7759/cureus.98848

**Published:** 2025-12-09

**Authors:** Jacob Gould, Saarang Patel, Bipin Chaurasia

**Affiliations:** 1 Krieger School of Arts and Sciences, Johns Hopkins University, Baltimore, USA; 2 Neurosurgery, Seton Hall University, South Orange, USA; 3 Neurosurgery, College of Medical Sciences, Bharatpur, NPL

**Keywords:** bibliometric analysis, epilepsy, epilepsy surgery, impact factor, neuromodulation, scholarly citations, temporal lobe epilepsy, top 100 most cited

## Abstract

Epilepsy surgery has undergone significant evolution over the past century, driven by advances in neuroimaging, neurophysiology, and surgical technique. As the volume of published research continues to expand, identifying the most influential studies is essential to understanding the development and current landscape of this field. A comprehensive search of the Scopus database was performed in March 2022 using a detailed Boolean strategy to identify publications related to epilepsy surgery. Articles were ranked by citation count, and the 100 most cited were analyzed for bibliometric parameters including citation frequency, impact factor, year of publication, geographic origin, journal distribution, authorship, and thematic focus. Data was compiled and analyzed using Microsoft Excel (Microsoft Corporation, Redmond, Washington, United States). The top 100 most cited articles accrued a total of 29,843 citations. Most were published between 1992 and 2001. The majority originated from the United States, with the most productive institutions being the University of California, Los Angeles (UCLA), the Cleveland Clinic, and Mayo Clinic. Temporal lobe epilepsy and resective surgery dominated the literature, followed by studies on neuromodulation, pediatric epilepsy, and advances in imaging and localization. This bibliometric review identifies the landmark studies that have shaped modern epilepsy surgery and underscores the pivotal role of temporal lobe epilepsy research and the emergence of neuromodulatory therapies. These findings provide a historical and thematic overview of the field and highlight emerging trends toward minimally invasive surgical innovations.

## Introduction and background

Epilepsy is one of the most prevalent chronic neurological disorders, which affects over 50 million individuals worldwide [1]. For patients with drug-resistant epilepsy, surgical intervention remains the most effective treatment option to achieve seizure control and improve quality of life [2,3]. Since the earliest resective procedures performed in the early 20th century, epilepsy surgery has evolved into a multidisciplinary field encompassing advances in neuroimaging, neurophysiology, neuronavigation, and minimally invasive techniques [4]. Over the past several decades, substantial research efforts have contributed to defining surgical indications, refining operative strategies, and improving postoperative outcomes.

The exponential growth of scientific output in epilepsy surgery has generated a vast and heterogeneous body of literature. Identifying the most influential contributions within this field is essential for understanding its development and guiding future research priorities. Bibliometric analysis provides a quantitative approach to assessing the academic influence and impact of scientific publications. Through citation-based metrics and publication trends, bibliometrics can help elucidate the evolution of research themes, collaboration networks, and institutional contributions for epilepsy surgery research. Furthermore, such analyses can assist clinicians and investigators in recognizing seminal works that have shaped current practice, while also highlighting underrepresented areas warranting further investigation [5].

The objective of this study was to identify and characterize the 100 most cited articles on epilepsy surgery. By conducting a comprehensive bibliometric review, we aimed to delineate the most impactful contributions to the field, analyze temporal and geographic publication trends, and provide insights into the progression of research and innovation in the surgical management of epilepsy.

## Review

Methods

In March 2022, the Scopus database was searched for all published articles related to epilepsy surgery. Scopus was selected for its broad coverage of biomedical and surgical journals compared with other databases. The search did not have time restrictions, and all original and review articles were included. To ensure comprehensive capture of the epilepsy surgery literature, a detailed Boolean search strategy was applied using the query outlined in Table 1.

**Table 1:** Search Strategy Structured search terms were applied using Scopus with title, abstract, and keyword fields (TITLE-ABS-KEY). Concepts included epilepsy surgery, epilepsy-related surgical procedures, and neuromodulatory interventions, combined using Boolean operators.

Concept	Key words search	Boolean logic
Epilepsy surgery	epilepsy surgery	OR
Epilepsy + Surgical procedures	epilepsy AND ( "resect*" OR "lesionectomy" OR "corpus callosotomy" OR "hemispherectomy" OR "hemispherotomy" OR "lobectomy" OR "cortical excision" OR "multiple subpial transection*" OR "posterior quadrant disconnection" OR "posterior quadrantectomy" OR "temporal parietal occipital disconnection" OR "temporal-parietal-occipital disconnection" OR "tempoparietooccipital disconnection" OR "radiosurgery" OR "gamma knife" )	OR
Neuromodulation	deep brain stimulation OR "vagal nerve stimulation" OR "vagus nerve stimulation" OR "responsive neurostimulation"	OR
Full combined strategy	TITLE-ABS-KEY ( "epilepsy surgery" OR ( "epilepsy" AND ( "resect*" OR "lesionectomy" OR "corpus callosotomy" OR "hemispherectomy" OR "hemispherotomy" OR "lobectomy" OR "cortical excision" OR "multiple subpial transection*" OR "posterior quadrant disconnection" OR "posterior quadrantectomy" OR "temporal parietal occipital disconnection" OR "temporal-parietal-occipital disconnection" OR "tempoparietooccipital disconnection" OR "radiosurgery" OR "gamma knife" OR "deep brain stimulation" OR "vagal nerve stimulation" OR "vagus nerve stimulation" OR "responsive neurostimulation" ) ) )	

These keywords were searched in the title, abstract, author, and keywords fields to ensure comprehensive inclusion of literature related to epilepsy surgery.

Only English-language articles focusing on the surgical management of epilepsy were included. Articles were screened for relevance by title, by abstract, and, as necessary, by full text. Publications not directly related to epilepsy surgery, such as those dealing exclusively with medical management or basic neuroscience without surgical correlation, were excluded. To minimize bias, two reviewers (SP and JG) independently conducted the search and screening. Both reviewers generated separate lists of the 100 most-cited articles, and discrepancies were resolved through discussion. When more than two articles had identical citation counts, they were assigned equal ranks with subsequent adjustments to the ranking order. The following variables were extracted for each article: (i) title, (ii) year of publication, (iii) authors, (iv) institution of first author, (v) country, (vi) journal, (vii) impact factor in 2019, (viii) total number of citations, (ix) average citations per year, (x) type of epilepsy, and (xi) type of surgery/intervention.

All data were compiled using Microsoft Excel (Microsoft Corporation, Redmond, Washington, United States) for descriptive analysis. Thematic categorization was performed manually.

Results

**Figure 1:**
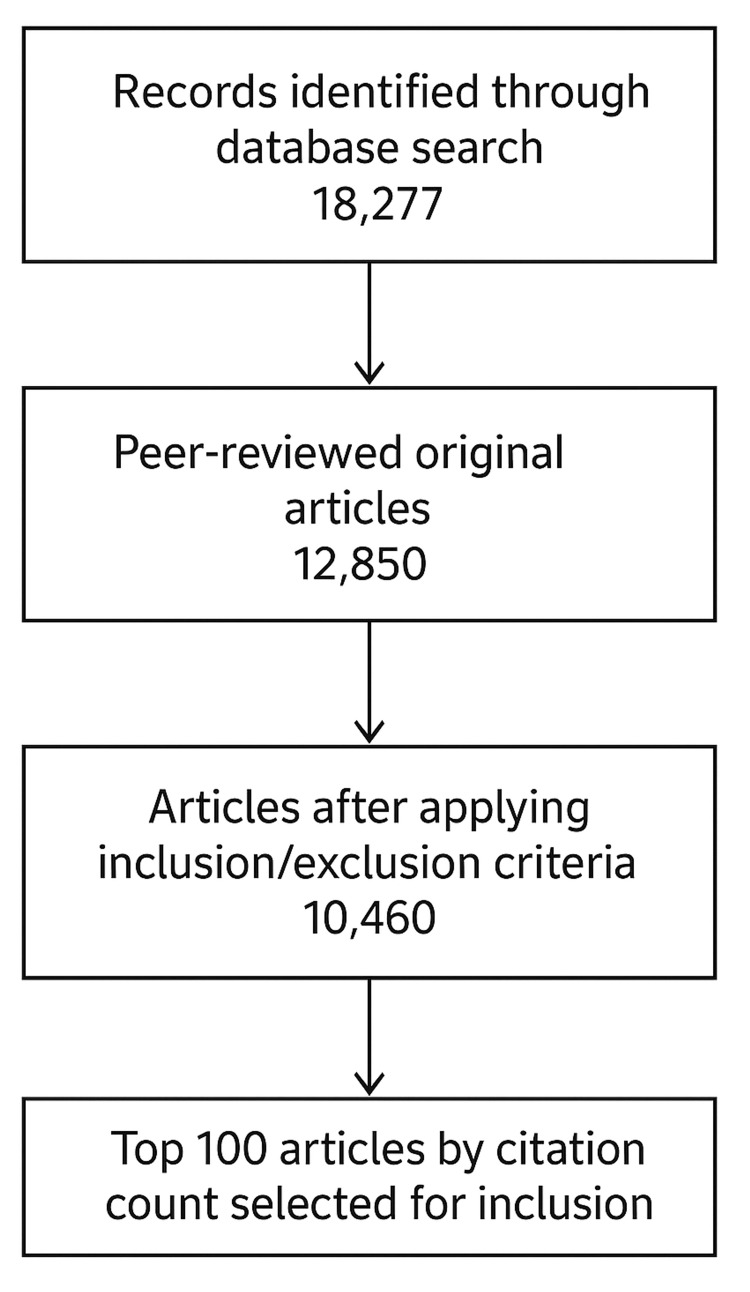
Search strategy

The initial database search yielded 18,277 records. Limiting results to peer-reviewed original research articles reduced the dataset to 12,850 articles. After applying predefined inclusion and exclusion criteria, 10,460 articles remained eligible for evaluation. From this pool, the top 100 most-cited articles were selected for full analysis based on citation count [6-105]. These results are summarized in Figure 1.

Collectively, the top 100 articles accumulated 29,843 citations with a mean of 298 citations per article and a median of 226.5 citations per article (range: 182-2237). The articles had an average 2019 impact factor of 10.77. The publication years for the top-cited papers demonstrate a clear surge in high-impact epilepsy surgery research beginning in the 1990s and peaking in the subsequent decade. The majority of these publications were produced between 1992 and 2001, with the greatest number of highly cited papers published during 1993. The most cited article was “A randomized, controlled trial of surgery for temporal-lobe epilepsy” by Wiebe et al., published in the *New England Journal of Medicine* in 2001 [6]. The top 10 cited papers are summarized in Table 2, with a complete list of the top 100 most cited papers provided in the Appendices. 

**Table 2:** Top 10 most cited articles on epilepsy surgery

Number of Citations	Article Title
2237	A randomized, controlled trial of surgery for temporal-lobe epilepsy [6]
962	Electrical stimulation of the anterior nucleus of thalamus for treatment of refractory epilepsy [7]
791	Vagus nerve stimulation therapy for partial-onset seizures: A randomized active-control trial [8]
737	Current concepts: Surgery for seizures [9]
648	Characteristics of medial temporal lobe epilepsy: I. Results of history and physical examination [10]
617	Early surgical therapy for drug-resistant temporal lobe epilepsy: A randomized trial [11]
506	Long-term treatment with vagus nerve stimulation in patients with refractory epilepsy [12]
487	A randomized controlled trial of chronic vagus nerve stimulation for treatment of medically intractable seizures [13]
469	Vagus Nerve Stimulation for Treatment of Partial Seizures: 1. A Controlled Study of Effect on Seizures [14]
456	Preoperative MRI predicts outcome of temporal lobectomy: An actuarial analysis [15]

**Figure 2:**
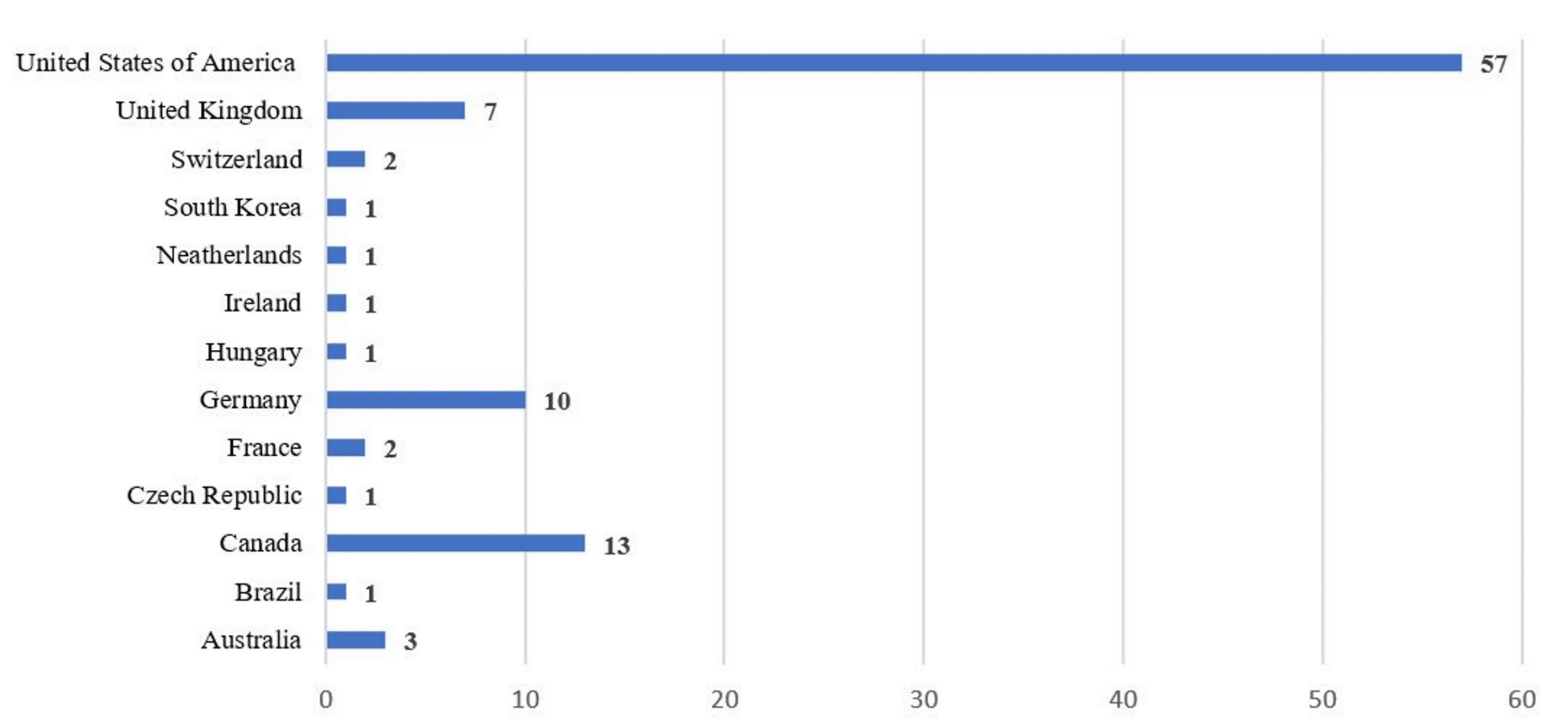
The 100 most cited articles distributed according to country of the first author

The 100 most cited articles were published across 29 journals. The journals with the highest number of publications were *Neurology* (n = 17), *Epilepsia* (n = 16), and *Annals of Neurology* (n = 13). Geographically, the majority of publications had first authors that originated from the United States (n = 57), Canada (n = 13), Germany (n = 10), and the United Kingdom (n = 7). The geographic breakdown is shown in Figure 2. First authors represented 64 different institutions, and the University of California, Los Angeles (UCLA) (n = 9), the Cleveland Clinic Foundation (n = 7), Mayo Clinic (n = 6), and McGill University (n = 6) were the most represented institutions in the dataset. 

The bibliometric analysis highlighted the contributions of a few highly prolific authors. The most frequently featured authors, each with a sustained impact over multiple decades, included Jerome Engel Jr. (n = 5), Hans Lüders (n = 5), and Gary Mathern (n = 5).

The analysis of surgical procedures within the top-cited literature reflected the historical and clinical dominance of resective surgery for temporal lobe epilepsy (TLE). Procedures focused on the temporal lobe, including temporal lobectomy and selective amygdalohippocampectomy, were the most frequently studied. Neuromodulation therapies also represented a significant segment of the highly-cited work with vagus nerve stimulation as the subject of eight papers (primarily originating from early randomized controlled trials in the 1990s). More advanced neuromodulation therapies, such as deep-brain stimulation (DBS) and responsive cortical stimulation, accounted for two papers. Finally, resective procedures for pediatric epilepsy, such as hemispherectomy, were highly discussed.

**Figure 3:**
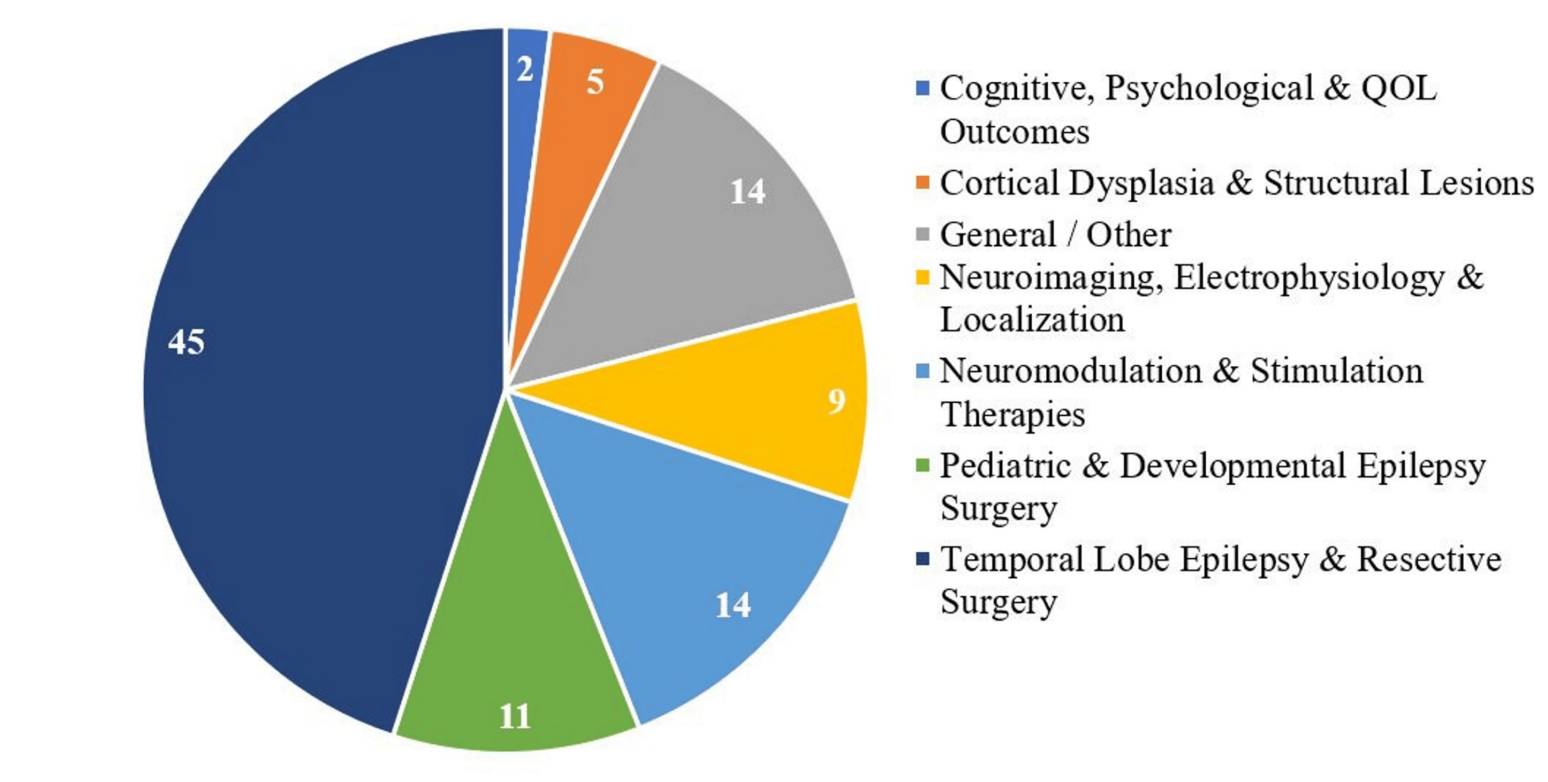
The top 100 most cited articles distributed according to thematic representation QOL: quality of life

Thematically, the most frequent topics among the top-cited papers centered around five key themes: (i) TLE and resective surgery outcomes, (ii) neuromodulation and stimulation-based therapies, (iii) pediatric and developmental epilepsy surgery, (iv) neuroimaging, electrophysiology, and localization techniques, and (v) cognitive, psychological, and quality of life outcomes. Fourteen papers did not fit within the primary thematic categories and were classified as “general/other”. These articles encompassed a range of topics, including historical overviews of epilepsy surgery, global access and healthcare disparities, cost-effectiveness and outcome prediction, and broad discussions of surgical principles, methodology, and ethical considerations. Figure 3 illustrates the distribution of research themes.

Discussion

This bibliometric analysis identifies and characterizes the 100 most cited articles in epilepsy surgery and offers insight into the historical trajectory and thematic evolution of this field. To our knowledge, this is the first bibliometric analysis focused exclusively on the most cited publications in epilepsy surgery. Previous bibliometric studies have examined the broader epilepsy literature, but few have delineated the specific impact of surgical research within this field [106]. The majority of highly cited studies originated from North America and were published between the 1990s and early 2000s, which corresponds to a period marked by major advances in neuroimaging, electrophysiological mapping, and microsurgical techniques [107]. This surge in impactful research reflects the maturation of epilepsy surgery as a scientific discipline and its transition from an experimental intervention to an evidence-based therapy.

Resective procedures for TLE dominated the most influential literature, reflecting the clinical centrality of mesial temporal sclerosis as a surgically remediable cause of epilepsy [108]. Landmark randomized trials, most notably that of Wiebe et al. (2001), firmly established the superiority of surgery over continued medical therapy in drug-resistant TLE [6]. Subsequent high-impact studies built on this foundation to refine patient selection, surgical approach, and long-term outcome assessment. The prominence of these works underscores how large, homogeneous patient populations and reproducible outcomes positioned TLE surgery as the cornerstone of modern epilepsy surgery research [6-105].

While temporal resections dominated early work, the emergence of neuromodulatory therapies, including vagus nerve stimulation (VNS), DBS, and responsive neurostimulation, introduced a new era of surgical innovation. These modalities expanded treatment to patients with multifocal or eloquent-region epilepsies who were previously poor surgical candidates [6-105]. Highly cited early VNS and DBS trials highlight the pivotal role of technological innovation in broadening surgical indications and fostering collaboration across disciplines. More recent literature increasingly emphasizes minimally invasive procedures such as laser interstitial thermal therapy and focuses on patient-centered outcomes, including cognition, psychosocial function, and quality of life [109,110]. This shift reflects a broader redefinition of surgical success from seizure control alone toward comprehensive well-being.

Geographically, North American centers, particularly UCLA, the Cleveland Clinic, Mayo Clinic, and McGill University, contributed disproportionately to the most cited work, illustrating the early concentration of epilepsy surgery expertise in a few specialized institutions [6-105]. However, the growing international representation in more recent years signals the potential global dissemination of epilepsy surgery programs and research, which may be aided by standardized protocols and consensus guidelines from organizations such as the International League Against Epilepsy (ILAE) [111]. Moving forward, the field is likely to continue evolving toward less invasive and technology-driven interventions.

Limitations

This study has several limitations inherent to bibliometric analyses. Citation counts may not fully capture the current clinical relevance or quality of individual studies, as they can be influenced by publication age, journal visibility, and self-citation practices. Restricting inclusion to the Scopus database and English-language articles may have excluded influential work published elsewhere. Finally, citation-based influence reflects academic impact rather than direct patient or societal benefit and should therefore be interpreted as one dimension of scholarly importance.

## Conclusions

This bibliometric review highlights the landmark studies that have defined the evolution of epilepsy surgery from early temporal lobectomy series to contemporary neuromodulation and imaging-guided techniques. The analysis underscores the dominance of temporal lobe surgery research and the rise of minimally invasive and stimulation-based interventions. Continued global collaboration and innovation will be essential to advance surgical care and improve outcomes for patients with drug-resistant epilepsy.

## Appendices

**Table 3:** Top 100 Most Cited Articles on Epilepsy Surgery

Rank	Number of Citations	Average Citations per year	Authors	Title	Year	Thematic Category	Journal	Impact Factor
1	2237	111.85	Wiebe S, Blume WT, Girvin JP, Eliasziw M	A randomized, controlled trial of surgery for temporal-lobe epilepsy [6]	2001	Temporal Lobe Epilepsy & Resective Surgery	New England Journal of Medicine	74.699
2	962	87.45	Fisher R, Salanova V, Witt T, et al.	Electrical stimulation of the anterior nucleus of thalamus for treatment of refractory epilepsy [7]	2010	Neuromodulation & Stimulation Therapies	Epilepsia	6.04
3	791	34.39	Handforth A, DeGiorgio CM, Schachter SC, et al.	Vagus nerve stimulation therapy for partial-onset seizures: A randomized active-control trial [8]	1998	Neuromodulation & Stimulation Therapies	Neurology	8.77
4	737	29.48	Engel J Jr	Current concepts: Surgery for seizures [9]	1996	General / Other	New England Journal of Medicine	74.699
5	648	23.14	French JA, Williamson PD, Thadani VM, Darcey TM, Mattson RH, Spencer SS, Spencer DD	Characteristics of medial temporal lobe epilepsy: I. Results of history and physical examination [10]	1993	Temporal Lobe Epilepsy & Resective Surgery	Annals of Neurology	9.037
6	617	68.5	Engel J Jr, McDermott MP, Wiebe S, et al.	Early surgical therapy for drug-resistant temporal lobe epilepsy: A randomized trial [11]	2012	Temporal Lobe Epilepsy & Resective Surgery	JAMA	18.652
7	506	23	Morris GL 3rd, Mueller WM	Long-term treatment with vagus nerve stimulation in patients with refractory epilepsy [12]	1999	Neuromodulation & Stimulation Therapies	Neurology	8.77
8	487	18.73	The Vagus Nerve Stimulation Study Group	A randomized controlled trial of chronic vagus nerve stimulation for treatment of medically intractable seizures [13]	1995	Neuromodulation & Stimulation Therapies	Neurology	8.77
9	469	17.37	Ben-Menachem E, Mañon-Espaillat R, Ristanovic R, et al.	Vagus Nerve Stimulation for Treatment of Partial Seizures: 1. A Controlled Study of Effect on Seizures [14]	1994	Neuromodulation & Stimulation Therapies	Epilepsia	6.04
10	456	17.54	Berkovic SF, McIntosh AM, Kalnins RM, et al.	Preoperative MRI predicts outcome of temporal lobectomy: An actuarial analysis [15]	1995	Temporal Lobe Epilepsy & Resective Surgery	Neurology	8.77
11	455	45.5	de Tisi J, Bell GS, Peacock JL, et al.	The long-term outcome of adult epilepsy surgery, patterns of seizure remission, and relapse: A cohort study [16]	2011	General / Other	The Lancet	60.392
12	448	17.23	Owen AM, Sahakian BJ, Semple J, Polkey CE, Robbins TW	Visuo-spatial short-term recognition memory and learning after temporal lobe excisions, frontal lobe excisions or amygdalo-hippocampectomy in man [17]	1995	Temporal Lobe Epilepsy & Resective Surgery	Neuropsychologia	2.652
13	436	15.57	Chelune GJ, Naugle RI, Lüders H, Sedlak J, Awad IA	Individual Change After Epilepsy Surgery: Practice Effects and Base-Rate Information [18]	1993	General / Other	Neuropsychology	2.477
14	433	18.83	Wyllie E, Comair YG, Kotagal P, Bulacio J, Bingaman W, et al.	Seizure outcome after epilepsy surgery in children and adolescents [19]	1998	Pediatric & Developmental Epilepsy Surgery	Annals of Neurology	9.037
15	405	36.82	Jacobs J, Zijlmans M, Zelmann R, et al.	High-frequency electroencephalographic oscillations correlate with outcome of epilepsy surgery [20]	2010	General / Other	Annals of Neurology	9.037
16	403	21.21	Hodaie M, Wennberg RA, Dostrovsky JO, Lozano AM	Chronic anterior thalamus stimulation for intractable epilepsy [21]	2002	Neuromodulation & Stimulation Therapies	Epilepsia	6.04
17	394	13.59	Jack CR Jr, Sharbrough FW, Cascino GD, Hirschorn KA, O'Brien PC, Marsh WR	Magnetic resonance image–based hippocampal volumentry: Correlation with outcome after temporal lobectomy [22]	1992	Temporal Lobe Epilepsy & Resective Surgery	Annals of Neurology	9.037
18	367	9.4	Wieser HG, Yaşargil MG	Selective amygdalohippocampectomy as a surgical treatment of mesiobasal limbic epilepsy [23]	1982	Temporal Lobe Epilepsy & Resective Surgery	Surgical Neurology	N/A
19	361	21.24	McIntosh AM, Kalnins RM, Mitchell LA, Fabinyi GC, Briellmann RS, Berkovic SF	Temporal lobectomy: Long-term seizure outcome, late recurrence and risks for seizure recurrence [24]	2004	Temporal Lobe Epilepsy & Resective Surgery	Brain	11.337
20	351	12.54	Williamson PD, French JA, Thadani VM, et al.	Characteristics of medial temporal lobe epilepsy: II. Interictal and ictal scalp electroencephalography, neuropsychological testing, neuroimaging, surgical results, and pathology [25]	1993	Temporal Lobe Epilepsy & Resective Surgery	Annals of Neurology	9.037
21	350	10.94	Morrell F, Whisler WW, Bleck TP	Multiple subpial transection: A new approach to the surgical treatment of focal epilepsy [26]	1989	General / Other	Journal of Neurosurgery	3.968
22	312	13.57	Radhakrishnan K, So EL, Silbert PL, Jack CR Jr, Cascino GD, Sharbrough FW, O'Brien PC	Predictors of outcome of anterior temporal lobectomy for intractable epilepsy: A multivariate study [27]	1998	Temporal Lobe Epilepsy & Resective Surgery	Neurology	8.77
23	309	11.88	Mathern GW, Babb TL, Vickrey BG, Melendez M, Pretorius JK	The clinical-pathogenic mechanisms of hippocampal neuron loss and surgical outcomes in temporal lobe epilepsy [28]	1995	Temporal Lobe Epilepsy & Resective Surgery	Brain	11.337
24	304.00	10.1	Awad IA, Rosenfeld J, Ahl J, Hahn JF, Lüders H	Intractable Epilepsy and Structural Lesions of the Brain: Mapping, Resection Strategies, and Seizure Outcome [29]	1991	Temporal Lobe Epilepsy & Resective Surgery	Epilepsia	6.04
25	299	23	Harvey AS, Cross JH, Shinnar S, Mathern GW	Defining the spectrum of international practice in pediatric epilepsy surgery patients [30]	2008	Pediatric & Developmental Epilepsy Surgery	Epilepsia	6.04
26	297	8.03	Spencer DD, Spencer SS, Mattson RH, Williamson PD, Novelly RA	Access to the posterior medial temporal lobe structures in the surgical treatment of temporal lobe epilepsy [31]	1984	Temporal Lobe Epilepsy & Resective Surgery	Neurosurgery	4.853
27	290	10.36	Trenerry MR, Jack CR Jr, Ivnik RJ, et al.	MRI hippocampal volumes and memory function before and after temporal lobectomy [32]	1993	Temporal Lobe Epilepsy & Resective Surgery	Neurology	8.77
28	283	20.21	Jeha LE, Najm I, Bingaman W, Dinner D, Widdess-Walsh P, Lüders H	Surgical outcome and prognostic factors of frontal lobe epilepsy surgery [33]	2007	General / Other	Brain	11.337
29	282	15.67	Devlin AM, Cross JH, Harkness W, Chong WK, Harding B, Vargha-Khadem F, Neville BG	Clinical outcomes of hemispherectomy for epilepsy in childhood and adolescence [34]	2003	Pediatric & Developmental Epilepsy Surgery	Brain	11.337
30	277	9.55	Vickrey BG, Hays RD, Graber J, Rausch R, Engel J Jr, Brook RH	A health-related quality of life instrument for patients evaluated for epilepsy surgery [35]	1992	Cognitive, Psychological & QOL Outcomes	Medical Care	3.21
31	275	9.48	Williamson PD, Thadani VM, Darcey TM, Spencer DD, Spencer SS, Mattson RH	Occipital lobe epilepsy: Clinical characteristics, seizure spread patterns, and results of surgery [36]	1992	General / Other	Annals of Neurology	9.037
32	266	6.65	Engel J Jr, Rausch R, Lieb JP, Kuhl DE, Crandall PH	Correlation of criteria used for localizing epileptic foci in patients considered for surgical therapy of epilepsy [37]	1981	General / Other	Annals of Neurology	9.037
33	265	9.46	Chugani HT, Shewmon DA, Shields WD, Sankar R, Comair Y, Vinters HV, Peacock WJ	Surgery for Intractable Infantile Spasms: Neuroimaging Perspectives [38]	1993	Pediatric & Developmental Epilepsy Surgery	Epilepsia	6.04
34	264	22	Lerner JT, Salamon N, Hauptman JS, et al.	Assessment and surgical outcomes for mild type i and severe type II cortical dysplasia: A critical review and the UCLA experience [39]	2009	Cortical Dysplasia & Structural Lesions	Epilepsia	6.04
35	262	7.28	Ojemann GA, Dodrill CB	Verbal memory deficits after left temporal lobectomy for epilepsy. Mechanism and intraoperative prediction [40]	1985	Temporal Lobe Epilepsy & Resective Surgery	Journal of Neurosurgery	3.968
36	260	13.68	Clusmann H, Schramm J, Kral T, et al.	Prognostic factors and outcome after different types of resection for temporal lobe epilepsy [41]	2002	Temporal Lobe Epilepsy & Resective Surgery	Journal of Neurosurgery	3.968
37	256	17.1	Cross JH, Jayakar P, Nordli D, Delalande O, Duchowny M, Wieser HG, Guerrini R, Mathern GW	Proposed criteria for referral and evaluation of children for epilepsy surgery: Recommendations of the subcommission for pediatric epilepsy surgery [42]	2006	Pediatric & Developmental Epilepsy Surgery	Epilepsia	6.04
38	251	11.41	Jokeit H, Ebner A	Long-term effects of refractory temporal lobe epilepsy on cognitive abilities: A cross-sectional study [43]	1999	Temporal Lobe Epilepsy & Resective Surgery	Journal of Neurology, Neurosurgery, and Psychiatry	8.234
39	246	8.48	Salanova V, Andermann F, Olivier A, Rasmussen T, Quesney LF	Occipital lobe epilepsy: Electroclinical manifestations, electrocorticography, cortical stimulation and outcome in 42 patients treated between 1930 and 1991: Surgery of occipital lobe epilepsy [44]	1992	Neuromodulation & Stimulation Therapies	Brain	11.337
40	242	12.7	Hertz-Pannier L, Chiron C, Jambaqué I, et al.	Late plasticity for language in a child's non-dominant hemisphere: A pre- and post-surgery fMRI study [45]	2002	Neuroimaging, Electrophysiology & Localization	Brain	11.337
41	242	8.1	Lévesque MF, Nakasato N, Vinters HV, Babb TL	Surgical treatment of limbic epilepsy associated with extrahippocampal lesions: The problem of dual pathology [46]	1991	Temporal Lobe Epilepsy & Resective Surgery	Journal of Neurosurgery	3.968
42	241	60.25	Blumcke I, Spreafico R, Haaker G, Coras R, Kobow K, et al	Histopathological findings in brain tissue obtained during epilepsy surgery [47]	2017	General / Other	New England Journal of Medicine	74.699
43	241	7.3	Hardiman O, Burke T, Phillips J, Murphy S, O'Moore B, Staunton H, Farrell MA	Microdysgenesis in resected temporal neocortex: Incidence and clinical significance in focal epilepsy [48]	1998	Temporal Lobe Epilepsy & Resective Surgery	Neurology	8.77
44	240	15	Spencer SS, Berg AT, Vickrey BG, et al.	Predicting long-term seizure outcome after resective epilepsy surgery: The multicenter study [49]	2005	Temporal Lobe Epilepsy & Resective Surgery	Neurology	8.77
45	239	17.1	Bowles B, Crupi C, Mirsattari SM, Pigott SE, Parrent AG, Pruessner JC, Yonelinas AP, Köhler S	Impaired familiarity with preserved recollection after anterior temporal-lobe resection that spares the hippocampus [50]	2007	Temporal Lobe Epilepsy & Resective Surgery	Proceedings of the National Academy of Sciences of the United States of America	9.412
46	237	9.48	Sperling MR, O'Connor MJ, Saykin AJ, Plummer C	Temporal lobectomy for refractory epilepsy [51]	1996	Temporal Lobe Epilepsy & Resective Surgery	JAMA	18.652
47	236	9.83	Behrens E, Schramm J, Zentner J, König R	Surgical and neurological complications in a series of 708 epilepsy surgery procedures [52]	1997	General / Other	Neurosurgery	4.853
48	232	8.92	Wyler AR, Hermann BP, Somes G	Extent of medial temporal resection on outcome from anterior temporal lobectomy: A randomized prospective study [53]	1995	Temporal Lobe Epilepsy & Resective Surgery	Neurosurgery	4.853
49	229	12.72	Sabsevitz DS, Swanson SJ, Hammeke TA, et al.	Use of preoperative functional neuroimaging to predict language deficits from epilepsy surgery [54]	2003	Neuroimaging, Electrophysiology & Localization	Neurology	8.77
50	228	14.25	Janszky J, Janszky I, Schulz R, Hoppe M, Behne F, Pannek HW, Ebner A	Temporal lobe epilepsy with hippocampal sclerosis: Predictors for long-term surgical outcome [55]	2005	Temporal Lobe Epilepsy & Resective Surgery	Brain	11.337
51	225	10.71	Sisodiya SM	Surgery for malformations of cortical development causing epilepsy [56]	2000	Cortical Dysplasia & Structural Lesions	Brain	11.337
52	223	7.43	Zatorre RJ, Jones-Gotman M	Human olfactory discrimination after unilateral frontal or temporal lobectomy [57]	1991	Temporal Lobe Epilepsy & Resective Surgery	Brain	11.337
53	222	14.8	Concha L, Gross DW, Wheatley BM, Beaulieu C	Diffusion tensor imaging of time-dependent axonal and myelin degradation after corpus callosotomy in epilepsy patients [58]	2006	Neuroimaging, Electrophysiology & Localization	NeuroImage	5.812
54	222	8.2	Valdueza JM, Cristante L, Dammann O, et al.	Hypothalamic hamartomas: With special reference to gelastic epilepsy and surgery [59]	1994	General / Other	Neurosurgery	4.853
55	221	17	Johnston JM, Vaishnavi SN, Smyth MD, et al.	Loss of resting interhemispheric functional connectivity after complete section of the corpus callosum [60]	2008	Neuroimaging, Electrophysiology & Localization	Journal of Neuroscience	5.673
56	221	6.69	Gabrieli JD, Cohen NJ, Corkin S	The impaired learning of semantic knowledge following bilateral medial temporal-lobe resection [61]	1988	Temporal Lobe Epilepsy & Resective Surgery	Brain and Cognition	2.508
57	219	5.92	Novelly RA, Augustine EA, Mattson RH, Glaser GH, Williamson PD, Spencer DD, Spencer SS, et al.	Selective memory improvement and impairment in temporal lobectomy for epilepsy [62]	1984	Temporal Lobe Epilepsy & Resective Surgery	Annals of Neurology	9.037
58	218	8.38	Chelune GJ	Hippocampal adequacy versus functional reserve: Predicting memory functions following temporal lobectomy [63]	1995	Temporal Lobe Epilepsy & Resective Surgery	Archives of Clinical Neuropsychology	2.226
59	215	23.89	Curry DJ, Gowda A, McNichols RJ, Wilfong AA	MR-guided stereotactic laser ablation of epileptogenic foci in children [64]	2012	Pediatric & Developmental Epilepsy Surgery	Epilepsy and Behavior	2.508
60	215	16.54	Sun FT, Morrell MJ, Wharen RE Jr	Responsive Cortical Stimulation for the Treatment of Epilepsy [65]	2008	Neuromodulation & Stimulation Therapies	Neurotherapeutics	6.035
61	215	13.44	Lee SK, Lee SY, Kim KK, Hong KS, Lee DS, Chung CK	Surgical outcome and prognostic factors of cryptogenic neocortical epilepsy [66]	2005	Cortical Dysplasia & Structural Lesions	Annals of Neurology	9.037
62	214	7.93	Ramsay RE, Uthman BM, Augustinsson LE, et al.	Vagus Nerve Stimulation for Treatment of Partial Seizures: 2. Safety, Side Effects, and Tolerability [67]	1994	Neuromodulation & Stimulation Therapies	Epilepsia	6.04
63	212	15.14	Wyllie E, Lachhwani DK, Gupta A, et al.	Successful surgery for epilepsy due to early brain lesions despite generalized EEG findings [68]	2007	Neuroimaging, Electrophysiology & Localization	Neurology	8.77
64	212	14.1	Cohen-Gadol AA, Wilhelmi BG, Collignon F, et al.	Long-term outcome of epilepsy surgery among 399 patients with nonlesional seizure foci, including mesial temporal lobe sclerosis [69]	2006	Temporal Lobe Epilepsy & Resective Surgery	Journal of Neurosurgery	3.968
65	208	17.3	Krsek P, Maton B, Jayakar P, et al.	Incomplete resection of focal cortical dysplasia is the main predictor of poor postsurgical outcome [70]	2009	Temporal Lobe Epilepsy & Resective Surgery	Neurology	8.77
66	206	8.96	Duchowny M, Jayakar P, Resnick T, et al.	Epilepsy surgery in the first three years of life [71]	1998	General / Other	Epilepsia	6.04
67	206	7.92	Salanova V, Andermann F, Rasmussen T, Olivier A, Quesney LF	Parietal lobe epilepsy clinical manifestations and outcome in 82 patients treated surgically between 1929 and 1988 [72]	1995	General / Other	Brain	11.337
68	205	8.2	Arruda F, Cendes F, Andermann F, et al.	Mesial atrophy and outcome after amygdalohippocampectomy or temporal lobe removal [73]	1996	Temporal Lobe Epilepsy & Resective Surgery	Annals of Neurology	9.037
69	202	8.42	Vining EP, Freeman JM, Pillas DJ, et al.	Why would you remove half a brain? The outcome of 58 children after hemispherectomy - The Johns Hopkins Experience 1968 to 1996 [74]	1997	Pediatric & Developmental Epilepsy Surgery	Pediatrics	2.69
70	202	8.08	Sawrie SM, Chelune GJ, Naugle RI, Lüders HO	Empirical methods for assessing meaningful neuropsychological change following epilepsy surgery [75]	1996	Cognitive, Psychological & QOL Outcomes	Journal of the International Neuropsychological Society	2.576
71	202	8.08	Hugg JW, Kuzniecky RI, Gilliam FG, Morawetz RB, Fraught RE, Hetherington HP	Normalization of contralateral metabolic function following temporal lobectomy demonstrated by 1H magnetic resonance spectroscopic imaging [76]	1996	Temporal Lobe Epilepsy & Resective Surgery	Annals of Neurology	9.037
72	199	12.44	Freitag H, Tuxhorn I	Cognitive function in preschool children after epilepsy surgery: Rationale for early intervention [77]	2005	Pediatric & Developmental Epilepsy Surgery	Epilepsia	6.04
73	199	6.63	Chelune GJ, Naugle RI, Lüders H, Awad IA	Prediction of cognitive change as a function of preoperative ability status among temporal lobectomy patients seen at 6-month follow-up [78]	1991	Temporal Lobe Epilepsy & Resective Surgery	Neurology	8.77
74	194	10.78	Wieser HG, Ortega M, Friedman A, Yonekawa Y	Long-term seizure outcomes following amygdalohippocampectomy [79]	2003	Temporal Lobe Epilepsy & Resective Surgery	Journal of Neurosurgery	3.968
75	193	9.19	Viskontas IV, McAndrews MP, Moscovitch M	Remote episodic memory deficits in patients with unilateral temporal lobe epilepsy and excisions [80]	2000	Temporal Lobe Epilepsy & Resective Surgery	Journal of Neuroscience	5.673
76	192	6.86	Pigott S, Milner B	Memory for different aspects of complex visual scenes after unilateral temporal- or frontal-lobe resection [81]	1993	Temporal Lobe Epilepsy & Resective Surgery	Neuropsychologia	2.652
77	191	7.64	Helmstaedter C, Elger CE	Cognitive consequences of two-thirds anterior temporal lobectomy on verbal memory in 144 patients: A three-month follow-up study [82]	1996	Temporal Lobe Epilepsy & Resective Surgery	Epilepsia	6.04
78	189	12.6	Andrade DM, Zumsteg D, Hamani C, Hodaie M, Sarkissian S, Lozano AM, Wennberg RA	Long-term follow-up of patients with thalamic deep brain stimulation for epilepsy [83]	2006	Neuromodulation & Stimulation Therapies	Neurology	8.77
79	189	6.75	Kuzniecky R, Burgard S, Faught E, Morawetz R, Bartolucci A	Predictive Value of Magnetic Resonance Imaging in Temporal Lobe Epilepsy Surgery [84]	1993	Temporal Lobe Epilepsy & Resective Surgery	Archives of Neurology	13.608
80	188	10.4	Delalande O, Fohlen M	Disconnecting surgical treatment of hypothalamic hamartoma in children and adults with refractory epilepsy and proposal of a new classification [85]	2003	Pediatric & Developmental Epilepsy Surgery	Neurologia Medico-Chirurgica	1.912
81	188	9.4	Adolphs R, Tranel D, Damasio H	Emotion recognition from faces and prosody following temporal lobectomy [86]	2001	Temporal Lobe Epilepsy & Resective Surgery	Neuropsychology	2.477
82	188	9.4	Funayama ES, Grillon C, Davis M, Phelps EA	A double dissociation in the affective modulation of startle in humans: Effects of unilateral temporal lobectomy [87]	2001	Temporal Lobe Epilepsy & Resective Surgery	Journal of Cognitive Neuroscience	3.105
83	188	6.48	Duchowny M, Levin B, Jayakar P, Resnick T, Alvarez L, Morrison G, Dean P	Temporal Lobectomy in Early Childhood [88]	1992	Temporal Lobe Epilepsy & Resective Surgery	Epilepsia	6.04
84	188	6.48	Theodore WH, Sato S, Kufta C, Balish MB, Bromfield EB, Leiderman DB	Temporal lobectomy for uncontrolled seizures: The role of positron emission tomography [89]	1992	Temporal Lobe Epilepsy & Resective Surgery	Annals of Neurology	9.037
85	187	11.69	Nimsky C, Ganslandt O, Hastreiter P, Wang R, Benner T, Sorensen AG, Fahlbusch R	Intraoperative diffusion-tensor MR imaging: Shifting of white matter tracts during neurosurgical procedures - Initial experience [90]	2005	Neuroimaging, Electrophysiology & Localization	Radiology	7.931
86	187	8.9	Paolicchi JM, Jayakar P, Dean P, Yaylali I, Morrison G, Prats A, Resnik T, Alvarez L, Duchowny M	Predictors of outcome in pediatric epilepsy surgery [91]	2000	Pediatric & Developmental Epilepsy Surgery	Neurology	8.77
87	187	8.5	Henry TR, Votaw JR, Pennell PB, Epstein CM, Bakay RA, Faber TL, Grafton ST, Hoffman JM	Acute blood flow changes and efficacy of vagus nerve stimulation in partial epilepsy [92]	1999	Neuromodulation & Stimulation Therapies	Neurology	8.77
88	187	6.68	Rausch R, Babb TL	Hippocampal Neuron Loss and Memory Scores Before and After Temporal Lobe Surgery for Epilepsy [93]	1993	Temporal Lobe Epilepsy & Resective Surgery	Archives of Neurology	13.608
89	186	26.6	Varadkar S, Bien CG, Kruse CA, Jensen FE, Bauer J, Pardo CA, Vincent A, Mathern GW, Cross JH	Rasmussen's encephalitis: Clinical features, pathobiology, and treatment advances [94]	2014	General / Other	The Lancet Neurology	30.039
90	186	6.89	Palmini A, Gambardella A, Andermann F, et al.	Operative Strategies for Patients with Cortical Dysplastic Lesions and Intractable Epilepsy [95]	1994	Cortical Dysplasia & Structural Lesions	Epilepsia	6.04
91	186	4.04	Davidson S, Falconer MA	Outcome of Surgery in 40 Children With Temporal-lobe Epilepsy [96]	1975	Temporal Lobe Epilepsy & Resective Surgery	The Lancet	60.392
92	184	8.75	Harden CL, Pulver MC, Ravdin LD, Nikolov B, Halper JP	A Pilot Study of Mood in Epilepsy Patients Treated with Vagus Nerve Stimulation [97]	2000	Neuromodulation & Stimulation Therapies	Epilepsy and Behavior	2.508
93	184	7.36	Wyllie E, Comair YG, Kotagal P, Raja S, Ruggieri P	Epilepsy surgery in infants [98]	1996	Pediatric & Developmental Epilepsy Surgery	Epilepsia	6.04
94	184	6.57	Cascino GD, Andermann F, Berkovic SF, et al.	Gelastic seizures and hypothalamic hamartomas: Evaluation of patients undergoing chronic intracranial EEG monitoring and outcome of surgical treatment [99]	1993	Neuroimaging, Electrophysiology & Localization	Neurology	8.77
95	184	6.34	Wyler AR, Curtis Dohan Jr F, Schweitzer JB, Berry III AD	A grading system for mesial temporal pathology (hippocampal sclerosis) from anterior temporal lobectomy [100]	1992	Temporal Lobe Epilepsy & Resective Surgery	Journal of Epilepsy	2.522
96	183	10.76	Fauser S, Schulze-Bonhage A, Honegger J, et al.	Focal cortical dysplasias: Surgical outcome in 67 patients in relation to histological subtypes and dual pathology [101]	2004	Cortical Dysplasia & Structural Lesions	Brain	11.337
97	183	9.15	Frost M, Gates J, Helmers SL, Wheless JW, Levisohn P, Tardo C, Conry JA	Vagus nerve stimulation in children with refractory seizures associated with Lennox-Gastaut syndrome [102]	2001	Neuromodulation & Stimulation Therapies	Epilepsia	6.04
98	182	14	Knowlton RC, Elgavish RA, Bartolucci A, et al.	Functional imaging: II. Prediction of epilepsy surgery outcome [103]	2008	Neuroimaging, Electrophysiology & Localization	Annals of Neurology	9.037
99	182	13	Zijlmans M, Huiskamp G, Hersevoort M, Seppenwoolde JH, van Huffelen AC, Leijten FS	EEG-fMRI in the preoperative work-up for epilepsy surgery [104]	2007	Neuroimaging, Electrophysiology & Localization	Brain	11.337
100	182	12.1	Tellez-Zenteno JF, McLachlan RS, Parrent A, Kubu CS, Wiebe S	Hippocampal electrical stimulation in mesial temporal lobe epilepsy [105]	2006	Neuromodulation & Stimulation Therapies	Neurology	8.77
